# IFN-*τ* Displays Anti-Inflammatory Effects on* Staphylococcus aureus* Endometritis via Inhibiting the Activation of the NF-*κ*B and MAPK Pathways in Mice

**DOI:** 10.1155/2017/2350482

**Published:** 2017-02-26

**Authors:** Zhenbiao Zhang, Yingfang Guo, Yuzhu Liu, Chengye Li, Mengyao Guo, Ganzhen Deng

**Affiliations:** Department of Clinical Veterinary Medicine, College of Veterinary Medicine, Huazhong Agricultural University, Wuhan 430070, China

## Abstract

The aim of the present study was to determine the anti-inflammatory effect of IFN-*τ* on endometritis using a mouse model of* S. aureus*-induced endometritis and to elucidate the mechanism of action underlying these effects. In the present study, the effect of IFN-*τ* on* S. aureus* growth was monitored by turbidimeter at 600 nm. IFN-*τ* did not affect* S. aureus* growth. The histopathological changes indicated that IFN-*τ* had a protective effect on uterus tissues with* S. aureus* infection. The ELISA and qPCR results showed the production of the proinflammatory cytokines TNF-*α*, IL-1*β*, and IL-6 was decreased with IFN-*τ* treatment. In contrast, the level of the anti-inflammatory cytokine IL-10 was increased. We further studied the signaling pathway associated with these observations, and the qPCR results showed that the expression of TLR2 was repressed by IFN-*τ*. Furthermore, the western blotting results showed the phosphorylation of I*κ*B, NF-*κ*B p65, and MAPKs (p38, JNK, and ERK) was inhibited by IFN-*τ* treatment. The results suggested that IFN-*τ* may be a potential drug for the treatment of uterine infection due to* S. aureus* or other infectious inflammatory diseases.

## 1. Introduction

Endometritis, which is a type of inflammation, can be caused by bacterial, viral, fungal, mycoplasma, and other infections or a combination of the above [1–3]. The clinical symptoms of endometritis include abnormal vaginal discharge and the dilation of the uterus and cervix. The diagnosis of subclinical cases requires the use of specific ultrasound examination, cytology, or biopsy [4]. The cause of endometritis is very complicated, and most studies have shown that an infection by pathogens is the major cause of endometritis [5].* Staphylococcus aureus (S. aureus)* is the most common pathogenic bacteria [6] and has been reported to induce both clinical and subclinical endometritis. Additionally, it can promote the production of proinflammatory cytokines by keratinocytes, triggering inflammatory disease [7].

Interferon-*τ* (IFN-*τ*) was originally identified as a pregnancy-associated protein in ruminants. It is produced by the trophoblast and is important for preventing the degradation of the corpus luteum. Additionally, it is also an early sign of female animal pregnancy [8, 9]. However, compared with the other known IFNs, IFN-*τ* also has high antiviral and antiproliferative activity but is not cytotoxic [10]. In contrast, the expression of related Type I IFN genes occurs in response to virus and other pathogens in a variety of inflammatory syndromes [11]. IFN-*τ* has a wide range of cross-species activity and functions in various cells, including lymphocytes, macrophages, and epithelial cells [12]. The specific function of IFN-*τ* is relatively unknown, but it has been shown to have antiviral activity [13]. IFN-*τ* has excellent antiviral activity, which is at least equivalent to that of IFN-*α* in the same species [14, 15]. IFN-*τ* has important implications in regulating cytokine networks, such as that involved in inflammation and angiogenesis, in the uterus during pregnancy [16]. Previous studies have shown that IFN-*τ* prevents the secretion of proinflammatory cytokines, thereby inhibiting* Candida albicans*-induced inflammation [17].

It is known that gram-negative and gram-positive bacteria are the most common microbial cause of inflammatory diseases [18].* S. aureus* is a well-known gram-positive bacteria, which can lead to inflammation from both community-acquired and nosocomial infections [19, 20].* S. aureus* is also the main pathogenic microbe that causes endometritis and enhances the expression of proinflammatory cytokines [21]. It has been shown IFN-*τ* has immunomodulatory properties [22]. A prominent feature of IFN-*τ* is its low cytotoxicity, compared with other Type I IFN, which has been observed in cell culture as well as in vivo [23]. In various clinical studies, IFN-*τ* has been shown to have anti-inflammatory properties and low cytotoxicity. As such, this study sought to verify the anti-inflammatory effect of IFN-*τ* in mice. Additionally, more studies on the functional role of IFN-*τ* in mediating anti-inflammatory effects are needed.

## 2. Materials and Methods

### 2.1. S. aureus Growth

*S. aureus* strain ATCC 25923 was cultured at 37°C in Mueller-Hinton II cation adjusted broth (MH, BD Biosciences, Sparks, MD, USA) 180 rpm with IFN-*τ* (Recombinant Ovine Interferon-tau, IFNT-29O, Creative Bioarray, New York, USA) treatment (0, 50, 100, and 200 ng/mL). The absorbance was measured once every 1 h at 600 nm until the bacteria reached the stationary phase.

### 2.2. Animals and Experiment Groups

Female BALB/c mice (6–8 weeks, 18–20 g) were purchased from the Center of Experimental Animals of Wuhan Institute. All experimental procedures were conducted with the approval of the Institutional Animal Care and were approved by the Huazhong Agricultural University Animal Care and Use Committee. The mice were housed in microisolator cages and received food and water ad libitum. Mice were given an adaptation period of 4–6 days prior to experimentation.

The sixty mice were randomly divided into six groups: (a) the control group (CG), in which the mice did not receive any treatment; (b) the* S. aureus*-infected group* (S. aureus)*, in which the mice were infected with* S. aureus* via an injection of 100 *μ*L* S. aureus*, into each uterine horn (a total volume of 200 *μ*L) through a microsyringe, followed by a stimulus of 24 h; (c) IFN-*τ* groups, in which the mice with* S. aureus* endometritis were treated with different concentrations of IFN-*τ* (2, 4, 8 mg/kg); and (d) the dexamethasone group (DEX), in which the mice with* S. aureus* endometritis were treatment with DEX (5 mg/kg, 5 mg/mL). Finally, all the mice were euthanized with sodium pentobarbital and sacrificed. The uterine tissues were quickly collected and were kept frozen at −80°C until they were used for subsequent experiments.

### 2.3. Histological Analysis

The harvested uterine tissues were fixed in 10% formaldehyde solution and embedded in paraffin wax. Sections (4 *μ*m) were deparaffinized with xylene and rehydrated through graded alcohols for staining. Then sections were stained with hematoxylin and eosin (H&E) and last examined with a microscope (Olympus, Japan).

### 2.4. Immunohistochemical Analysis

Six serial sections of each uterus area were collected and mounted on polylysine coated slides. A 4 *μ*m section was prepared from the paraffin-embedded block and dehydrated and decreasing concentrations of ethanol and then washed for 5 min in running water. Following washing, sections were treated with 90% formic acid for 5 mins and blocked for endogenous peroxidase activity in 5% H_2_O_2_. A Tris-HCl (0.05 M, pH 7.66) was introduced at room temperature for 20 min and with a 1 : 200 dilution (*v*/*v*) of the MPO antibody (Abcam) was added to the tissues and incubated at 4°C overnight. As for the negative control, the primary antibody was replaced with PBS. The secondary antibodies (Tianjin Sungene Biotech Co., Ltd. China) were added as appropriate and 3,3′-diaminobenzidine staining was visualized using the hematoxylin stain. Two pathologists scored the slides, respectively. For MPO staining assessment, mounting with glycerin and observing used a microscope with a camera system (Olympus DP-71, MN, USA).

### 2.5. HEK293 Cell Culture and Transfection

HEK293 cells were purchased from American Type Culture Collection (ATCC, Manassas, VA, USA) and were cultured in DMEM containing 10% FBS with 5% CO_2_ at 37°C. The culture media were changed once every 24 h. The cells were always added 1 h prior to* S. aureus* treatment and were subsequently incubated in the presence or absence of various concentrations of IFN-*τ*. HEK293 cells were transfected with pEGFP-N1-mTLR2 plasmids using FuGENE HD transfection reagent, according to the manufacturer's instructions (Roche Applied Science, Indianapolis, IN, USA).

### 2.6. ELISA Analysis

The uterus samples were weighed and homogenized with phosphate-buffered saline (*w/v*, 1 : 9) on ice and then centrifuged at 12000 rpm for 15 min at 4°C. The supernatant was collected. The cytokine TNF-*α*, IL-1*β*, IL-6, and IL-10 assays were evaluated with the corresponding ELISA kits (Biolegend, USA) according to the instructions of the manufacturer. Results are expressed as means ± SD of three independent experiments.

### 2.7. Quantitative Real-Time Polymerase Chain Reaction

Total RNA from endometrial samples were isolated using TRIzol reagent (Invitrogen Corporation) according to manufacturer's instructions. The concentration and purity of the total RNA were determined spectrophotometrically at 260/280 nm. The RNA was reverse transcribed into cDNA using a Revert Aid First Strand cDNA Synthesis Kit (Thermo). The primers (TNF-*α*, IL-1*β*, IL-6, IL-10, and TLR2) used for qPCR are listed in Table 1 (Designed by software Primer 5.0). The qPCR were executed by a 7500 Fast Real-Time PCR System (Applied Biosystems) with the SYBR green Plus reagent kit (Roche Applied Science, Mannheim, Germany) in a 20 *μ*L system. Each sample set three repetitions. Results were expressed as 2^−ΔΔCt^ comparative method. ΔΔCt = (target gene Ct of experimental group − reference gene Ct of experimental group) − (target gene Ct of control group − reference gene Ct of control group). GAPDH was used as the reference gene.

### 2.8. Western Blot Analysis

The protein of collected tissue samples were extracted using PMSF (10 *μ*L) and RIPA (1 mL) function 30 min. Then protein concentration was determined using the BCA protein assay kit (Thermo, USA). The crude protein per sample was subjected to 10% SDS-PAGE using Tris-HCl Precast Gels and subsequently transferred onto PVDF membranes. The membrane was blocked with Tris buffer saline containing 0.05% Tween-20 (TBS-T), supplemented with 5% skim milk at room temperature for 1.5 h on a rotary shaker, following by TBS-T washed. The membranes were washed with a specific primary antibody (Cell Signaling Technology Inc., Danvers, MA) diluted in TBS-T containing skim milk (*v*/*v* = 1 : 1000) at 4°C overnight. Subsequently, the membranes were washed with TBS-T. The membrane was washed and treated with secondary antibody (Tianjin Sungene Biotech Co., Ltd., Tianjin, China) diluted in TBS-T (*v*/*v* = 1 : 5000) at room temperature for 2 h. In the last step, membranes developed with the ECL Plus Western Blotting Detection System (Amersham Life Science, UK). *β*-Actin (Tianjin, China) was used as a control.

### 2.9. Statistical Analysis

Statistical analyses of the data were performed using the SPSS software (ver. 17 for Windows; SPSS Inc., Chicago, IL, USA). All values are expressed as the means ± SD. The data were assessed using the Tukey-Kramer method for multiple comparisons. Significance was determined by a 1-way ANOVA using a significance level of *p* < 0.05.

## 3. Results

### 3.1. Effect of IFN-*τ* on* S. aureus* Growth

**Figure 1:**
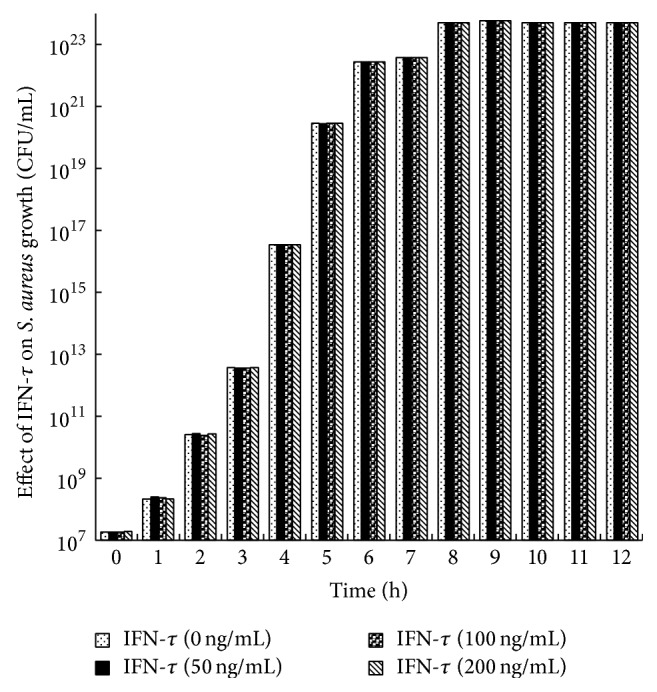
Effect of IFN-*τ* on* S. aureus* growth.* S. aureus* (1.8 × 10^7^ CFU/mL) was cultured in MH broth supplemented with different concentrations of IFN-*τ* (50–200 ng/mL) in each of the experiments.* S. aureus* growth was monitored turbidimetrically (600 nm) after 12 h. The data represents the mean of triplicates ± SD from three independent experiments.

The effect of IFN-*τ* (50–200 ng/mL) on* S. aureus* growth was monitored by turbidimeter at 600 nm for 12 h. The results showed that IFN-*τ* had no effect on* S. aureus* growth. Specifically, when compared to the untreated controls,* S. aureus* grown in the presence of IFN-*τ* (50–200 ng/mL) were not affected (Figure 1).

### 3.2. Histopathological Changes

**Figure 2:**
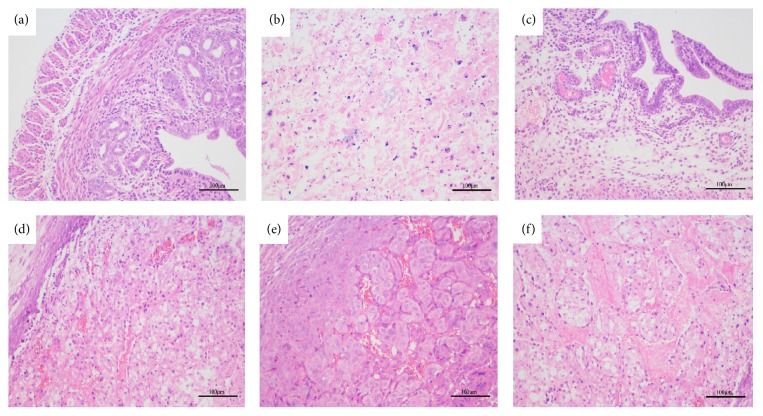
Histopathological changes (HE, ×200). H&E staining of the harvested uterine tissues. (a) Control group (CG), in which the mice were not treated. (b)* S. aureus*-infected group* (S. aureus)*, in which the mice were infected with* S. aureus*. (c) Dexamethasone group (DEX), in which the mice inoculated with* S. aureus* and developed endometritis were treated with DEX. (d)–(f) IFN-*τ* groups, in which the mice inoculated with* S. aureus* and developed endometritis were treated with different concentrations of IFN-*τ* (2, 4, 8 mg/kg).

Tissues were collected 24 hours after the injection of* S. aureus* and were stained with hematoxylin and eosin (H&E). There were no pathological changes in the uterine tissue of the CG (Figure 2(a)). The uterine morphology of the mice inoculated with* S. aureus* displayed inflammatory cell infiltration, endometrial congestion, severe destruction of the myometrium cells, and the disappearance of the uterus acinar structure (Figure 2(b)). However, IFN-*τ* significantly reduced the inflammation stimulated by* S. aureus*, and the uterine tissue structure of the mice treated with IFN-*τ* was almost complete, with only minor endometrial pathology and an insignificant amount of inflammatory cell infiltration (Figures 2(c), 2(d), and 2(e)). Representative pictures are shown to demonstrate the effects dose-dependent effects of IFN-*τ*. The degree of inflammatory injury was also very slight in the DEX group, as shown in Figure 2(f).

### 3.3. Effects of IFN-*τ* on MPO Activity in Uterus Tissues

**Figure 3:**
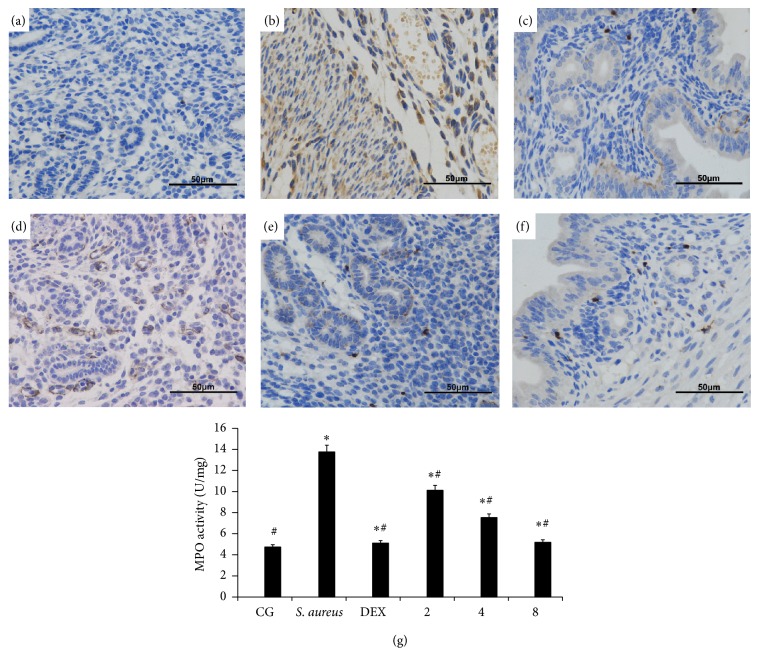
Effects of IFN-*τ* on MPO activity in uterus tissues (HE, ×200). Immunohistochemical illustrates MPO activity in sections of uterine tissues. (a) Control group (CG), the mice were without any treatment. (b)* S. aureus*-infected group* (S. aureus)*, the mice were infected with* S. aureus.* (c) Dexamethasone group (DEX), the mice of* S. aureus* endometritis were treatment with DEX. (d)–(f) IFN-*τ* groups, the mice of* S. aureus* endometritis were treatment with different concentrations of IFN-*τ* (2, 4, 8 mg/kg). (g) Effects of different doses of IFN-*τ* (2, 4, 8 mg/kg) and dexamethasone (5 mg/kg) on myeloperoxidase activity in mice with* S. aureus*-infected uterine tissues. Data are expressed as the means ± SD (*n* = 10). ^*∗*^*p* < 0.05, significantly different from the CG; ^#^*p* < 0.05, significantly different from the* S. aureus* group.

MPO activity has been used as a quantitative index of inflammation and can illustrate the level of neutrophil infiltration. The effect of IFN-*τ* on MPO activity was examined in mice with* S. aureus*-induced endometritis. Immunohistochemical staining showed that MPO specific immunolabelling was scarcely found in the neutrophil and mononuclear cells of lamina propria of mucosa in normal uterus in CG (Figure 3(a)), whereas this expression was found elevated in cells of surface epithelium and in cells of the inflammatory infiltrate in* S. aureus*-infected group (Figure 3(b)). The MPO activity was very slight in DEX group as shown in Figure 3(c). In contrast, mice treated with different doses of IFN-*τ* uterus showed a lower level of MPO activity, the immunoreactivity of which showed the suppressive effects of IFN-*τ* increased in a dose-dependent manner in uterus samples of mice (Figures 3(d), 3(e), and 3(f)). The activity of MPO from different mice groups was also measured by MPO assay kits. The results illustrates that the activity of MPO was low in CG and dramatically increased in* S. aureus* group (Figure 3(g)), while the* S. aureus*-infected mice treated with different concentrations of IFN-*τ* (2, 4, 8 mg/kg) significantly inhibited the activity of MPO (compare with* S. aureus* group). The results indicated that reduction of neutrophil infiltration in uterine tissues could be one mechanism underlying the protective effect of IFN-*τ*.

### 3.4. Effects of IFN-*τ* on Inflammatory Cytokines

**Figure 4:**
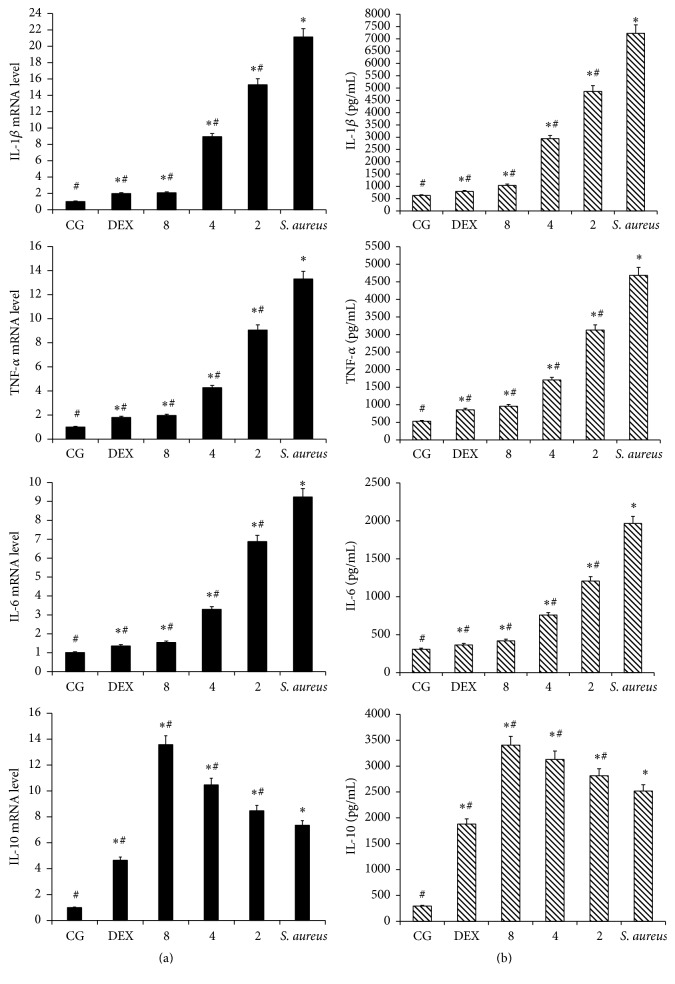
Cytokine concentrations. (a) TNF-*α*, IL-1*β*, IL-6, and IL-10 mRNA levels in the uterine tissue. (b) TNF-*α*, IL-1*β*, IL-6, and IL-10 protein levels in the uterine tissue. The data represent the contents of 1 mL supernatant of uterine homogenate and are presented as the means ± SD (*n* = 10). The CG refers to the control group,* S. aureus* refers to the mice with* S. aureus*-induced endometritis that were not given any drug treatment, 2, 4, and 8 refer to the IFN-*τ* (2, 4, and 8 mg/kg) administration groups, and DEX refers to the dexamethasone treatment group. ^*∗*^*p* < 0.05, significantly different from the CG; ^#^*p* < 0.05, significantly different from the* S. aureus* group.

To determine the effect of IFN-*τ* on the* S. aureus* endometritis, the levels of TNF-*α*, IL-1*β*, IL-6, and IL-10 were measured by qPCR and ELISA. The results are shown in Figure 4. The expressions of TNF-*α*, IL-1*β*, and IL-6 were increased significantly in the mice inoculated with* S. aureus*, compared to the CG. The increases in TNF-*α*, IL-1*β*, and IL-6 expression, which were induced by* S. aureus*, were significantly inhibited by IFN-*τ*. Compared with the mice inoculated with* S. aureus*, the mRNA and protein expression of TNF-*α*, IL-1*β*, and IL-6 was significantly decreased following IFN-*τ* treatment. The effects of IFN-*τ* increased in a dose-dependent manner. With increasing concentrations of IFN-*τ*, more significant effects were observed. However, the effect on IL-10 was different. The mRNA and protein expression of IL-10 was increased in the mice inoculated with* S. aureus* compared to the CG. Compared with the mice inoculated with* S. aureus*, the expression of IL-10 further increased upon IFN-*τ* treatment. With increasing concentrations of IFN-*τ*, more significant effects on IL-10 were observed.

### 3.5. Effects of IFN-*τ* on TLR2 Expression

**Figure 5:**
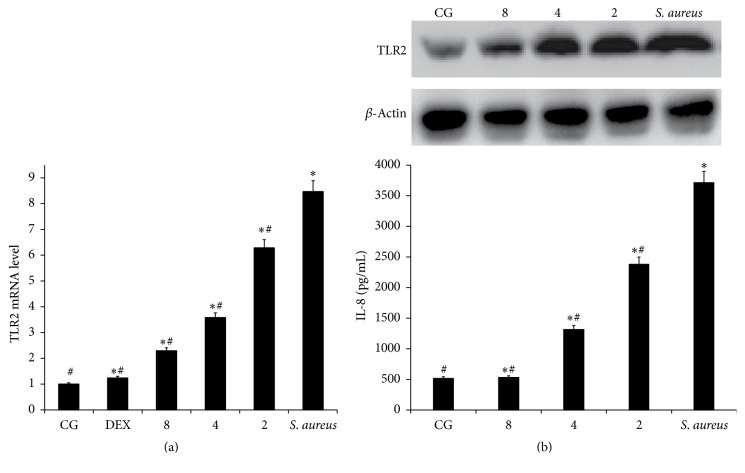
Effects of IFN-*τ* on TLR2. (a) The TLR2 mRNA levels in the uterine tissues. (b) The TLR2 and IL-8 protein levels in HEK293-mTLR2 cells. qPCR was performed to assess the mRNA levels of TLR2. Western blotting was performed to detect the protein levels of TLR2. *β*-Actin was used as a control. The CG refers to the control group,* S. aureus* refers to the mice with* S. aureus*-induced endometritis that were not given any drug treatment, 8, 4, and 2 refer to the IFN-*τ* (2, 4, and 8 mg/kg) administration groups, and DEX refers to the dexamethasone treatment group. ^*∗*^*p* < 0.05, significantly different from the CG; ^#^*p* < 0.05, significantly different from the* S. aureus* group.

TLR2 signaling plays an important role in the innate inflammatory response. The secretion of TLR2 by* S. aureus*-infected can induce the activation of the NF-*κ*B and MAPK pathways and subsequently result in the release of proinflammatory cytokines. Compared to the CG, TLR2 mRNA levels were significantly increased in the* S. aureus* group. The results showed that IFN-*τ* had a suppressive effect on the expression of TLR2 mRNA, which was originally upregulated by* S. aureus* in the uterine tissues (Figure 5(a)).

To further confirm that IFN-*τ* inhibited the inflammatory response via TLR2, the effect of IFN-*τ* on the production of IL-8 in* S. aureus*-stimulated HEK293-mTLR2 cells was assessed. The results showed that IFN-*τ* inhibited the expression of TLR2 and IL-8 (Figure 5(b)) in* S. aureus*-stimulated HEK293-mTLR2 cells. With increasing doses of IFN-*τ*, the effect became increasingly significant.

### 3.6. IFN-*τ* Affected the Activation of the NF-*κ*B Pathway

**Figure 6:**
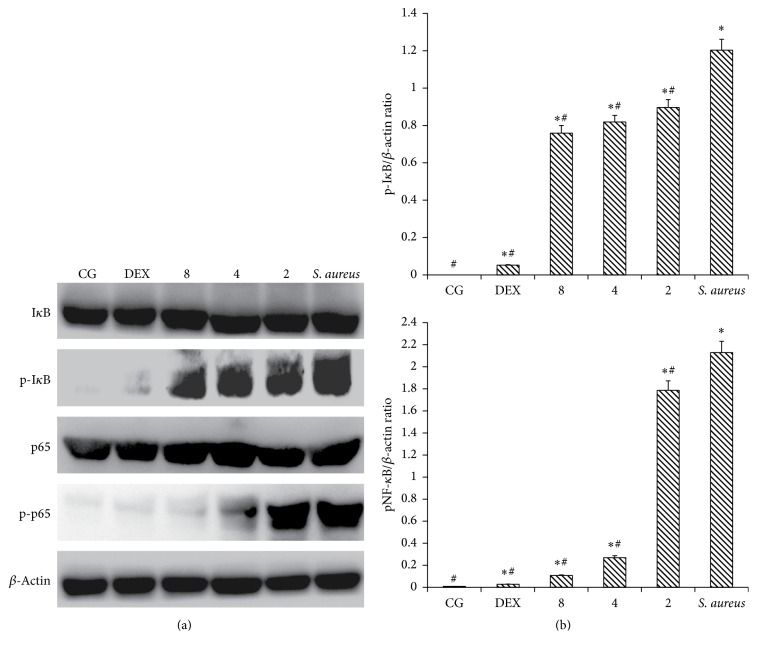
Effect of IFN-*τ* on NF-*κ*B activation. (a) The NF-*κ*B and I-*κ*B protein levels in uterine tissues. (b) The ratio of phosphorylation proteins levels as well as *β*-actin levels. Western blot was performed to detect the levels of total and phosphorylated NF-*κ*B and I-*κ*B proteins in the control group (CG), the* S. aureus*-induced endometritis without drug treatment group* (S. aureus)*, and the IFN-*τ* administration groups (2, 4, 8 mg/kg). *β*-Actin was used as a control. The values are presented as the means ± SD (*n* = 10). ^*∗*^*p* < 0.05, significantly different from the CG; ^#^*p* < 0.05, significantly different from the* S. aureus* group.

NF-*κ*B is an important signaling molecule in the development of inflammatory diseases. Once activated, NF-*κ*B induces the production of proinflammatory cytokines. In the present study, we examined NF-*κ*B activation by western blot analysis. As shown in Figure 6, the phosphorylation of the I*κ*B*α* and p65 proteins was activated in mice inoculated with* S. aureus* but not in CG. The phosphorylation level in mice inoculated with* S. aureus* was significantly higher than that in the IFN-*τ* treatment groups. These observations indicate that IFN-*τ* can suppress the activation of NF-*κ*B through the inhibition of the phosphorylation of I*κ*B*α* and p65. Compared to the mice inoculated with* S. aureus*, the level of phosphorylation was significantly reduced with increasing IFN-*τ* concentrations in the treatment groups. The results showed that IFN-*τ* can inhibit NF-*κ*B I*κ*B*α* and p65 phosphorylation in a dose-dependent manner.

### 3.7. IFN-*τ* Inhibits the Activation of the MAPK Pathway

**Figure 7:**
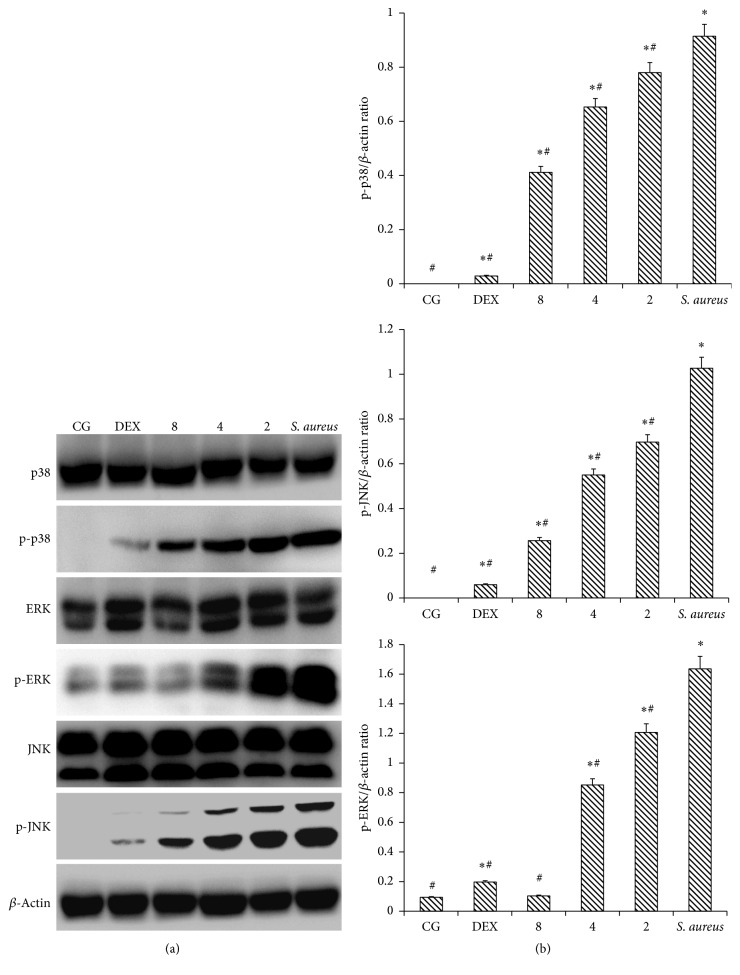
Effect of IFN-*τ* on MAPK activation. (a) The protein levels of MAPKs in uterine tissues. (b) The ratio of phosphorylation proteins as well as *β*-actin levels. Western blotting was performed to measure the levels of total and phosphorylated p38, ERK, and JNK protein in the Control group (CG), the* S. aureus*-induced endometritis without drug treatment group* (S. aureus)*, and the IFN-*τ* administration groups (2, 4, and 8 mg/kg). *β*-Actin was used as a control. The values are presented as the means ± SD (*n* = 10). ^*∗*^*p* < 0.05, significantly different from the CG; ^#^*p* < 0.05, significantly different from the* S. aureus* group.

MAPK is an important signaling molecule in the development of inflammatory diseases. The activation of p38, JNK and ERK can induce MyD88-dependent signaling pathways, which, in turn, induce MAPK activation. We found that* S. aureus*-infected uterine tissues showed increased phosphorylation of three MAPKs. In the mice inoculated with* S. aureus*, the phosphorylation levels of p38, ERK, and JNK were significantly increased, compared with CG (Figure 7). Additionally, the phosphorylation levels of the three MAPKs in the mice inoculated with* S. aureus* were significantly higher than in the mice treated with IFN-*τ*. These data indicate that IFN-*τ* can suppress the phosphorylation of p38, JNK and ERK. Compared to the mice inoculated with* S. aureus*, in the treatment groups, the level of phosphorylation was reduced significantly with increasing concentrations of IFN-*τ*. These results showed that IFN-*τ* can inhibit MAPK p38, JNK, and ERK phosphorylation in a dose-dependent manner.

## 4. Discussion

IFN-*τ*, a novel Type I IFN, is secreted by the trophoblast ectoderm of ruminants in the early stages of pregnancy [22]. IFN-*τ* regulates the expression of numerous genes in the endometrium and has been shown to play an important biological role in the implantation stage of pregnancy [24]. Endometritis is a local inflammatory condition, which delays uterine degeneration and has a significant economic impact on dairy production [25, 26]. However, infection with microbes, such as* S. aureus*, can cause the destruction of the uterine horn structure, subsequently resulting in the loss in the ability to conceive. This has been found to be due to inflammation and necrosis, as determined by studies utilizing morphological and image measurement tools [27–29]. In the present study, based on histopathological observations, we found that IFN-*τ* has anti-inflammatory functions as well as a significant protective effect on uterine tissues following infection with* S. aureus*.

A key component of the host immune response to infection is the upregulation of cytokine production [30]. To further study the effect of IFN-*τ* on* S. aureus* endometritis, the levels of TNF-*α*, IL-1*β*, IL-6, and IL-10 production were evaluated. The results showed that the levels of TNF-*α*, IL-1*β*, and IL-6, which are induced by* S. aureus*, were significantly inhibited by IFN-*τ* treatment in a dose-dependent manner. During the inflammatory response, TNF-*α* is induced by regulated genes acting as effective inducers and subsequently regulates the expression of cytokines, chemokines, and cell adhesion molecules [31, 32]. In vivo, TNF-*α* can activate the intracellular I*κ*B*α* and JNK signaling pathways through TLRs, thus contributing to the production of proinflammatory cytokines [33]. The proinflammatory cytokine, IL-1*β*, is a key regulator of acute inflammatory processes in the central nervous system [34, 35]. Pathogen-associated molecular patterns of* S. aureus* can activate inflammatory pathways, and when bound to innate monocytes/macrophages, they result in a significant increase in the secretion of TNF-*α* and IL-6 [36, 37]. IL-6 is a pleiotropic cytokine that regulates multiple biological processes, including the development of inflammation, immune responses, and the acute phase reaction [38]. IL-10 has been shown to be beneficial in improving systemic immunity by having anti-inflammatory effects and promoting the repair of immune dysfunction after the invasion of different pathogens [39]. Our experimental results showed that the expression of IL-10 mRNA and protein was increased in mice inoculated with* S. aureus*, and the expression of IL-10 was further increased with IFN-*τ* treatment. With increasing concentrations of IFN-*τ*, the effect on IL-10 was more significant. IFN-*τ* can promote the secretion of inflammatory cytokines, including IL-10, thereby inhibiting the subsequent secretion of TNF-*α*, IL-1*β*, and IL-6 [40]. TLRs are transmembrane pattern recognition receptors, which are part of the innate immune system and are the key element in identifying viral and bacterial components [37, 41]. TLR2 senses the inflammatory reactions caused by* S. aureus* most rapidly and sensitively and presents pathogen-derived antigens to various types of cells [42]. Our results showed TLR2 increased in mice infected with* S. aureus*, and its secretion was significantly reduced after IFN-*τ* treatment. TLR2 plays a key role in the response to both innate and adaptive immunity, as it can identify exogenous pathogen-associated molecular patterns (PAMPs) and induce proinflammatory signaling pathways [43]. In this study, IFN-*τ* inhibited the secretion of TLR2 in a dose-dependent manner.

NF-*κ*B and MAPKs are two important inflammation-associated pathways, and they can modulate the development of inflammation by mediating downstream signaling networks [44]. To further investigate the mechanism by which IFN-*τ* affects inflammation, we analyzed the NF-*κ*B and MAPK pathways. The NF-*κ*B signaling pathway is involved in the regulation of genes that mediate inflammation, as well as various cytokines, and is associated with numerous chronic inflammatory diseases [45]. Normally, the NF-*κ*B p65 subunit is bound to I*κ*B in the cytosol. When stimulated by various pathogens, NF-*κ*B p65 is released due to the phosphorylation of I*κ*B, leading to its activation and nuclear translocation, which then triggers inflammation [46]. Western blotting analysis showed the phosphorylation levels of I*κ*B*α* and p65 in mice infected with* S. aureus* were significantly higher than that in the IFN-*τ* groups. These results indicated that IFN-*τ* can inhibit the phosphorylation of I*κ*B*α* and p65, thereby blocking NF-*κ*B activation. In addition to NF-*κ*B, MAPKs are another key regulator of signal transduction pathways that can modulate the levels of inflammatory mediators [47]. Specifically, p38, JNK, and ERK are the major effectors of the MAPK pathway. The phosphorylation of these three proteins can cause the translocation of Activator Protein-1 (AP-1) to the nucleus, thereby promoting the inflammatory response [48, 49]. In the current study, we found an increase in the phosphorylation levels of p38, JNK, and ERK in the mice inoculated with* S. aureus*. However, in the groups treated with IFN-*τ*, the phosphorylation of these proteins was significantly inhibited. Recent studies have indicated that the inhibition of phosphorylation of MAPKs with specific inhibitors could significantly attenuate inflammation by decreasing the number of neutrophils. Similar effects were observed following the inhibition of proinflammatory cytokines and chemokines [50]. In our study, we demonstrated that IFN-*τ* significantly inhibited the phosphorylation of proteins involved in MAPK pathway activation. Taken together, our results showed that blocking the NF-*κ*B and MAPK signaling pathways and inhibiting the secretion of proinflammatory cytokines are the main mechanisms by which IFN-*τ* abates endometritis.

## 5. Conclusion

Our study showed that* S. aureus* increases the expression of TNF-*α*, IL-1*β*, and IL-6, thereby activating TLR2 signaling pathways, including NF-*κ*B and MAPK. The experiments presented herein show that IFN-*τ* reduces the secretion of proinflammatory cytokines, such as TNF-*α*, IL-1*β*, and IL-6, while promoting the production of the anti-inflammatory cytokine IL-10. This reduction inhibits inflammation and enhances the repair of uterine tissues. In addition, IFN-*τ* inhibited the expression of TLR2, thereby blocking the activation of the NF-*κ*B and MAPK signaling pathways and abating endometritis. Additional studies are required to validate the efficacy of IFN-*τ* as a treatment for uterine infection due to* S. aureus* or other infectious pathogens.

## Figures and Tables

**Table 1:** Sequences of primers used for qPCR.

Gene	GenBank accession number	Sequence (5′-3′): sense and antisense	Product size (bp)
TNF-*α*	NM_013693.3	Sense: 5′-CTTCTCATTCCTGCTTGTG-3′	198
		Antisense: 5′-ACTTGGTGGTTTGCTACG-3′	
IL-1*β*	NM_008361.3464	Sense: 5′-AGGCTCCGAGATGAACAA-3′	464
		Antisense: 5′-AAGGCATTAGAAACAGTCC-3′	
IL-6	NM_031168.1	Sense: 5′-TTCTTGGGACTGATGCTG-3′	380
		Antisense: 5′-CTGGCTTTGTCTTTCTTGTT-3′	
IL-10	NM_010548.2	Sense: 5′-AACATACTGCTAACCGACTC-3′	286
		Antisense: 5′-TGGCCTTGTAGACACCTT-3′	
TLR2	NM_011905.3	Sense: 5′-TTTGCTCCTGCGAACTCC-3′	267
		Antisense: 5′-GCCACGCCCACATCATTC-3′	
GAPDH	NM_001289726.1	Sense: 5′-TGTTTCCTCGTCCCGTAG-3′	108
		Antisense: 5′-CAATCTCCACTTTGCCACT-3′	
